# Dr. John H. Charnley: An Architect and Pioneer of the Modern Era of Hip Replacement Surgery

**DOI:** 10.7759/cureus.68832

**Published:** 2024-09-06

**Authors:** Mukesh O Phalak, Ajinkya K Chaudhari, Tushar Chaudhari, Anteshwar Birajdar

**Affiliations:** 1 Department of Orthopaedics, Dr. D. Y. Patil Medical College, Hospital and Research Centre, Dr. D. Y. Patil Vidyapeeth (Deemed to be University), Pune, IND

**Keywords:** arthroplasty, biographical memoir, dr john h charnley, hip reconstructive surgery, historical vignette, john charnley, pioneer, scientific legacy, tribute

## Abstract

Dr. John H. Charnley (1924-1982) revolutionized orthopedic surgery with his groundbreaking innovations in hip replacement with exceptional skill and a holistic thought process, which has had an impact to this day in the world of arthroplasty. His innovations have improved the lives of numerous patients who had painful and discomforting arthritis and have been instrumental in providing painless mobile joints to these patients. This article reviews Charnley's contributions to the development of low-friction arthroplasty using ultra-high-molecular-weight polyethylene and the use of acrylic bone cement for improved implant fixation. These advancements dealt with the critical issues of friction, wear, and implant stability, significantly enhancing patient outcomes and implant longevity. Charnley’s work led to the global standardization of hip replacement procedures, influencing orthopedic practices globally and setting benchmarks for modern implant designs. His principles continue to inform ongoing research and advancements in hip replacement technology. This review also discusses the challenges and criticisms faced by Charnley’s innovations, reflecting on their evolution and impact on contemporary orthopedic surgery. A surgeon blessed with a noble heart who would help his patients who were in trouble by going out of the way and was determined for a better tomorrow, self-driven by his compassion and ambition for treating his patients. Charnley's legacy remains pivotal in shaping the field and improving the quality of life for patients undergoing hip replacement surgery.

## Introduction and background

Dr. John H. Charnley, born on August 29, 1911, stands as a towering figure in the history of orthopedic surgery, particularly renowned for his pioneering work in hip replacement surgery. His innovations transformed the treatment of hip disorders and significantly improved the quality of life for countless patients. He was a man of compassion and ambition for a better tomorrow, with a noble intention of solving the problems of his patients. He started with the goal of finding a long-term, definitive cure for crippling arthritis of the hip joint. During this journey, he discovered numerous problems but, like a true clinician of perseverance, worked hard to solve each one of them. He had an excellent understanding of biological factors and mechanical factors playing a role in the disease, which then enabled him to pave the way for groundbreaking work in the world of arthroplasty. Furthermore, Charnley has contributed insignificantly to techniques of closed reduction through his book “The Closed Treatment of Common Fractures,” which has gone through multiple editions and reprints and is currently in its centenary edition [1]. This detailed article explores Charnley’s life, his groundbreaking contributions to hip replacement, and the enduring impact of his work on modern orthopedics.

## Review

Early life and education

**Figure 1:**
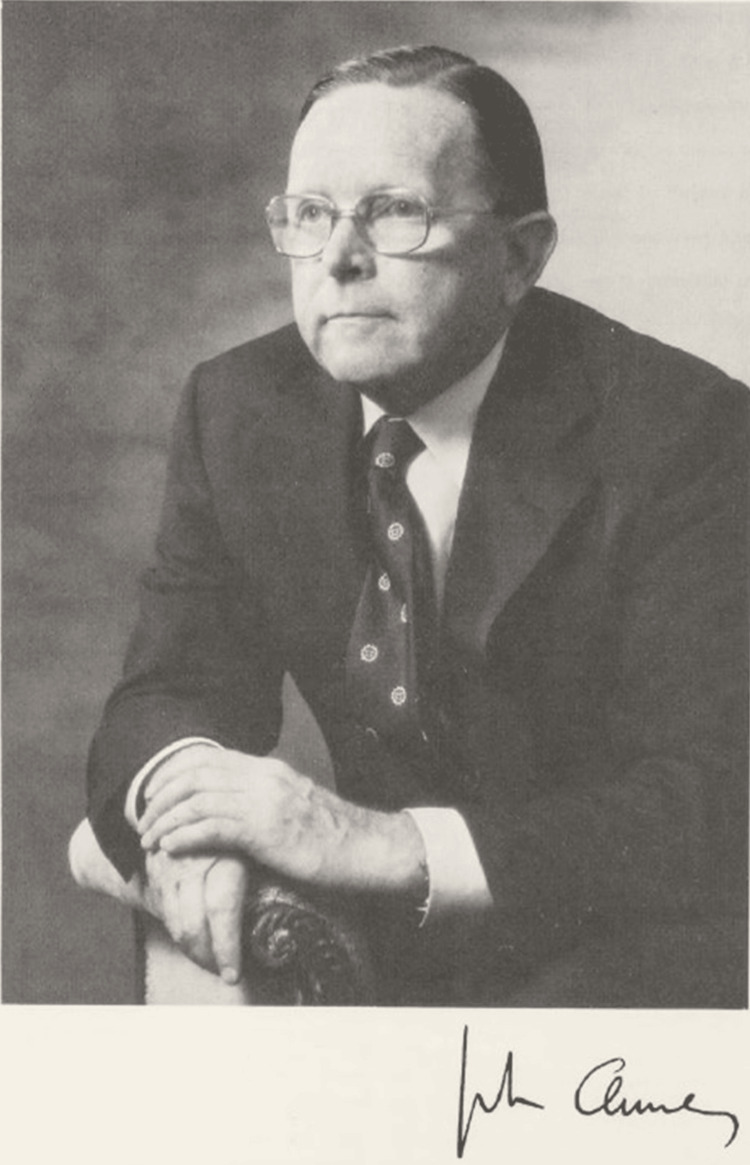
Dr. John H. Charley A portrait of the arthroplasty legend Dr. John H. Charnley along with his graceful autograph. Image Credit: Nisbet and Woodruff (open-access article) [4]

Charnley was born in England on August 29, 1911, to Arthur Walker Charnley, a pharmacist, and Lily Hodgson, a nurse by profession [2]. His early years were marked by an interest in both engineering and medicine, which would later converge in his groundbreaking work in orthopedic surgery. He even was a member of the local Liberal Club in his earlier years. After completing his pre-medical studies at St. John’s College, Cambridge, he passed the examination for a fellowship of the Royal College of Surgeons of England in 1936. He was appointed as a house surgeon at the Manchester Royal Infirmary, followed by a Resident Surgical Officer at the Salford Royal Hospital. He also spent some time researching traumatic shock at King’s College, London [3]. Charnley was encouraged to specialize in orthopedics by Professor Harry Platt, the chair of orthopedics at Manchester University. He also served in the Royal Army Medical Corps as an orthopedic specialist in 1939. He participated in the war at Dunkirk and developed the Charnley caliper, an adjustable modification of the Thomas walking caliper that enabled an injured soldier to get away from the battlefield and was instrumental in saving numerous lives. Charnley returned to Manchester University as a lecturer under Prof. Harry Platt and, in 1947, became the consultant orthopedic surgeon to the Manchester Royal Infirmary. His journey in the field of hip surgeries started in 1963. He even built a biomechanical laboratory at the Manchester Infirmary. In 1972, Charnley became the Chair of Orthopedic Surgery at Manchester University and retired as Professor Emeritus in 1976 (Figure 1). This eminent personality departed for a heavenly abode on August 5, 1982 [4].

Pre-Charnley era in hip surgery

Before Charnley’s innovations, hip replacement surgery was fraught with challenges. The earliest step toward arthroplasty was by simply trimming the articular ends of bones in tubercular conditions or stiff joints. Early attempts at hip arthroplasty often led to unsatisfactory outcomes due to high rates of infection, dislocation, and implant failure. Surgeons used various materials, including metal-on-metal components and simple plaster casts, which were less effective and durable. The lack of standardized techniques and materials contributed to inconsistent results and limited the procedure’s applicability.

Charnley’s innovations and contributions

Charnley’s work began to revolutionize hip replacement surgery in the 1960s. His contributions can be categorized into several key areas.

Surgeries of Hip Joint

Charnley worked extensively on surgeries of the hip joint. He wrote a paper titled “Surgery of the Hip Joint,” which is a record of the lecture given to the East Denbigh and Flint Division of the British Medical Association on March 26, 1959 [5]. This paper highlighted topics like basic surgeries of a degenerated hip joint, limitations of arthroplasty, techniques and outcomes of arthrodesis of the hip joint, displacement osteotomy of the hip joint, central dislocation stabilization and its arthrodesis, arthrodesis of the hip in difficult pathologies and possibilities, and future of hip arthroplasty, and penned the benefits and views on a low-friction prosthesis and design of the Charnley prosthesis. This paper is a milestone in the world of orthopedics and hip joint surgeries.

Low-Friction Arthroplasty

**Figure 2:**
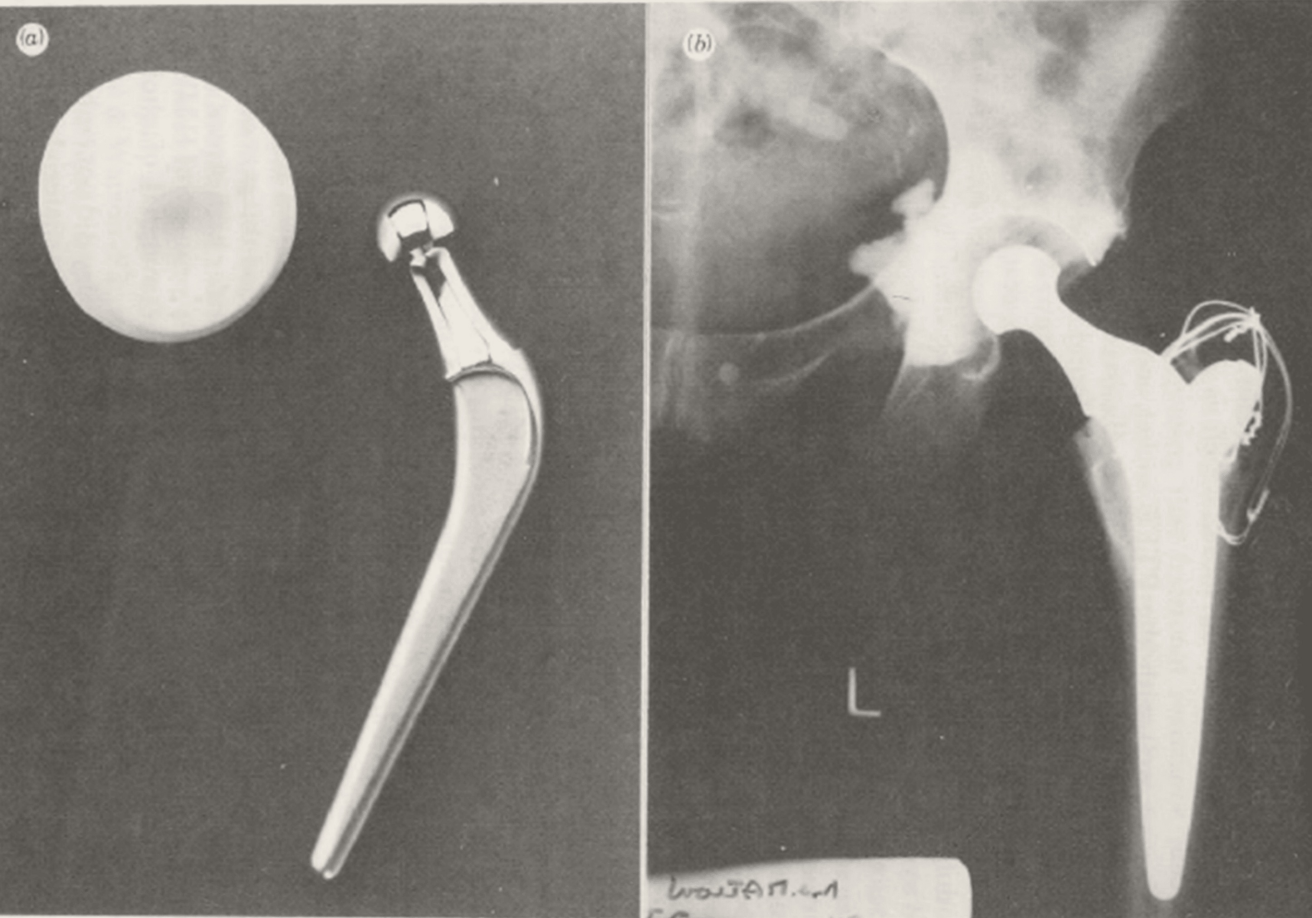
Dr. John H. Charnley's design of hip prosthesis (a) Charnley's design of low-friction arthroplasty prosthesis, (b) radiograph of the implanted prosthesis Image Credit: Charnley (open-access article) [6]

Charnley’s most significant contribution was the development of low-friction arthroplasty (Figure 2) [6]. He understood that excessive friction between the hip implant components led to increased wear, eventual implant failure, and the need for repeat surgery. Charnley introduced the use of ultra-high-molecular-weight polyethylene (UHMWPE) for the acetabular component to tackle the issue of wear and tear to better long-term outcomes [7]. This material’s low-friction properties significantly reduced wear compared to earlier materials. Harry Craven demonstrated the use of a wear-testing rig with the positive outcome of reduced wear. The UHMWPE liner, in combination with a polished metal femoral head, allowed for smoother joint movement and improved the longevity of the implant. Charnley also wrote a book titled “Low-Friction Arthroplasty of the Hip: Theory and Practice” [8]. This book talks about the theory of frictional torque in total hip arthroplasty, biomechanics of hip joints, use of small-diameter heads, use of high-molecular-weight polyethylene on metal, and method of reattachment of the trochanter. It can be safely said that the work of Charnley has been penned very comprehensively, laid the foundation for future research, and paved the path for advancements in hip arthroplasty.

Cemented Hip Prosthesis

Another critical innovation by Charnley was the use of self-curing acrylic bone cement, specifically polymethylmethacrylate (PMMA), to create a strong bond within minutes between the elements of the prosthesis and the patient’s bone to secure the prosthetic components [9]. This cemented fixation method improved the stability and fixation of the implant, reducing the likelihood of loosening and improving overall implant longevity. The use of PMMA allowed for better integration of the implant with the bone, and the exothermic reaction using heat to kill any germs and organisms reduced infection, leading to more predictable and successful outcomes. Charnley used this fixation method as a standard step in the procedure of arthroplasty, marking an important milestone.

Design and Material Selection

Charnley’s design philosophy emphasized simplicity and functionality. His hip prosthesis featured a smooth, spherical femoral head and a cup-shaped acetabular component designed to mimic the natural anatomy of the hip joint. The choice of materials, including stainless steel for the femoral stem and UHMWPE for the acetabular cup, was based on both mechanical and biological considerations. These materials provided the necessary strength and biocompatibility, contributing to the overall success of the implants.

Biomechanical Insights

Charnley’s work extended beyond material and design innovations to include a deep understanding of joint biomechanics. He meticulously studied the forces acting on the hip joint and designed his implants to accommodate these forces effectively. Charnley concluded the length of levers through which different muscles act should be equal, and to achieve this medialization of the femoral head to adjust the center of motion is important. Based on the understanding of biomechanical factors, Charnley incorporated steps like corrective osteotomy for fixed external rotation, lateral displacement of the great trochanter with detachment of soft tissue, and acetabular reaming. The insights into biomechanics also informed the design of implants that could withstand the stresses of normal activity, enhancing their durability and performance.

Clean Air in the Operating Room

Charnley also worked on the concept of clean air in the operating room. The work was published in 1973 and highlighted key concepts to prevent intraoperative infection due to airborne bacteria like laminar airflow in an operating system, aspirators for the nose and mouth, ideal cloth material for operating gowns, use of foam pressure pads, diagrammatic demonstration of the advantage of laminar airflow, the idea of using a hood, which Charnley called a headpiece with a transparent window and suction holes during arthroplasty, and appropriate gown design and position. These findings are of prime importance to date to prevent surgical site infection [10,11].

Closed Treatment of Common Fractures

Charnley wrote a book titled “The Closed Treatment of Common Fractures,” which has gone through multiple reprints and is currently in the centenary edition [1]. This book highlights important topics like compression arthrodesis and the use of spring-loaded clamps to compress bony surfaces. The book is important in the world of orthopedics; it has the techniques and essence of the non-operative approach to fracture management. It also enlightens the junior orthopedic surgeon with a simple approach and the importance of minor details while managing fractures conservatively.

Impact of Charnley

The impact of Charnley’s work can be measured in several ways:

Reduction in Postoperative Complications

The introduction of low-friction arthroplasty and cemented implants led to a marked reduction in complications such as dislocation, infection, and implant loosening. Patients experienced fewer adverse events and better overall outcomes, contributing to the growing popularity of hip replacement surgery. A prospective study of 102 patients with Charnley prostheses showed good outcomes with fewer complications [12,13].

Increased Longevity of Implants

Charnley’s designs significantly improved the longevity of hip implants. The reduced friction and improved fixation allowed patients to enjoy a better quality of life for many years after surgery. This advancement in implant durability has had a lasting impact on the field of orthopedic surgery.

Standardization of Hip Replacement Procedures

Charnley’s methods provided a standardized approach to hip replacement surgery that facilitated training for surgeons and improved the predictability of outcomes. The establishment of standardized techniques contributed to the widespread acceptance and success of hip replacement procedures.

Global Influence

Charnley’s work had a profound influence on orthopedic practices worldwide. His techniques and designs were adopted and refined by surgeons across the globe, leading to widespread improvements in hip replacement surgery. Charnley’s innovations became a benchmark in the field, shaping the development of modern hip implants and surgical techniques.

Legacy and Influence

Charnley’s contributions to orthopedic surgery extend far beyond his own time. His work laid the foundation for many broad advancements in hip replacement and orthopedic surgery. The Charnley Hip Prosthesis remains a benchmark in the field, and his principles continue to inform modern implant designs and surgical techniques. Charnley’s work has been integral to the education of orthopedic surgeons. His publications, including the seminal text "Low-Friction Arthroplasty of the Hip: Theory and Practice," have been essential resources for understanding hip arthroplasty. These texts have guided generations of surgeons and provided a foundation for further research and development in the field. Charnley’s innovations prompted ongoing research into improving hip replacement technology. Modern advancements, including minimally invasive techniques and new materials, build upon the principles established by Charnley. Researchers continue to explore ways to enhance implant materials, design, and surgical techniques, reflecting the enduring impact of Charnley’s work. Throughout his career, Charnley received numerous accolades and honors for his contributions to medical science (Table 1). He was recognized by professional societies and received awards acknowledging his pioneering work in hip replacement surgery. His legacy is celebrated in various forms, including the naming of awards and institutions in his honor.

**Table 1:** Awards and recognition earned by Dr. John H. Charnley C.B.E.: Commander of the Order of the British Empire, F.R.S.: Fellow of the Royal Society

Dr. John H. Charnley: An Orthopedic Legend	
Honors	
1970	C.B.E.
1974	Freeman of the Borough of Bury
1975	F.R.S.
1977	Knight Bachelor Emeritus Professor of Orthopaedic Surgery, University of Manchester
Honorary Degrees and Fellowships	
1972	Honorary Fellow, American College of Surgeons
1976	Honorary M.D., University of Liverpool
1977	Honorary M.D., University of Uppsala, Sweden
1978	Honorary D.Sc., University of Leeds Honorary M.D., Queen’s University, Belfast Honorary Fellow, American Academy of Orthopaedic Surgery
1981	Honorary Fellow, British Orthopaedic Association
Medals, Prizes, and Orations	
1969	Olaf Af Acrel Medal of the Swedish Surgical Society
1971	Prix Mondial Nessim Habif of the University of Geneva Gold Medal of the Society of Apothecaries of London Lawrence Poole Prize of the University of Edinburgh
1972	McMurray Memorial Lecture, University of Liverpool Cecil Joll Prize, Royal College of Surgeons of England
1973	Wade Professor, Royal College of Surgeons of Edinburgh Gairdner Foundation Annual Award
1974	Lasker Foundation Award
1975	Cameron Prize, University of Edinburgh Lister Medal and Oration, Royal College of Surgeons of England
1976	Robert Jones Lecturer, British Orthopaedic Association Prince Philip Gold Medal Award of the Plastics and Rubber Institute Prize Buccheri La Ferla
Honorary and Foreign Memberships	
American Orthopaedic Association	
Belgian Orthopaedic Association	
Brazilian Orthopaedic Association	
Finnish Orthopaedic Association	
French Orthopaedic Association	
French-Canadian Orthopaedic Association	
Scandinavian Orthopaedic Association	

Criticisms and challenges

Despite his remarkable contributions, Charnley’s work faced criticisms and challenges. In the initial stages of his work, some patients experienced complications such as implant loosening and wear. However, these issues were addressed through continuous refinement of his designs and techniques. Charnley’s commitment to improving his work led to better outcomes and fewer complications over time. While UHMWPE was a significant advancement, it was not without its limitations, such as wear over time. This issue has led to ongoing research into alternative materials and improved designs. Researchers continue to explore new materials and technologies to address the challenges associated with wear and longevity. As with any pioneering work, Charnley’s methods have undergone adaptations and modifications. Some variations of his original designs have been developed to address specific patient needs and advancements in technology. While these adaptations have contributed to improvements in hip replacement surgery, they also reflect the ongoing evolution of the field.

## Conclusions

Charnley’s contributions to orthopedic surgery, particularly in the field of hip replacement, seeded a transformative era in joint replacement. His innovations in low-friction arthroplasty, cemented implants, and joint biomechanics have left a lasting impact on the field, improving the lives of countless patients. Charnley’s work not only advanced surgical techniques but also set the stage for ongoing research and development in hip replacement technology. His legacy continues to influence orthopedic surgery, and the principles remain central to modern practices. This article provides a comprehensive exploration of Charnley’s life and work, highlighting his significant contributions to modern orthopedic surgery and his enduring legacy.
